# Motor circuits and beyond: Functional connectivity related to psychomotor syndromes in depression

**DOI:** 10.1017/S0033291725101852

**Published:** 2025-09-29

**Authors:** Qunjun Liang, Zhifeng Zhou, Ying Li, Shengli Chen, Shiwei Lin, Xiaoshan Lin, Yingli Zhang, Bo Peng, Ziyun Xu, Gangqiang Hou, Yingwei Qiu

**Affiliations:** 1 Department of Medical Imaging, Shenzhen Nanshan People’s Hospital, Shenzhen University, Shenzhen, PR China; 2 School of Marxism, Guangxi University of Science and Technology, Liuzhou, PR China; 3 Neuropsychiatry Imaging Center, Department of Radiology, Shenzhen Mental Health Center, Shenzhen Kangning Hospital, Shenzhen, PR China; 4 Department of Radiology, General Hospital of Ningxia Medical University, Yinchuan, PR China; 5 Department of Radiology, The Tenth Affiliated Hospital, Southern Medical University (Dongguan People’s Hospital), Dongguan, PR China; 6 Department of Depressive Disorders, Shenzhen Kangning Hospital, Shenzhen Mental Health Center, Shenzhen, PR China

**Keywords:** functional magnetic resonance, major depressive disorder

## Abstract

**Background:**

Psychomotor disturbance (PmD) is prevalent in major depressive disorder (MDD), with neural substrates implicated in disrupted motor circuits and the interaction to non-motor cortex. Our objective is to explore the functional connectivity pattern underlying PmD using functional magnetic resonance imaging (fMRI).

**Methods:**

A total of 150 patients with MDD and 91 healthy controls (HCs) were included in this study. The patients were categorized into psychomotor (pMDD, n = 107) and non-psychomotor (npMDD, n = 43) groups based on the Hamilton Depression Rating Scale. Seed-based connectivity (SBC) analysis was conducted using predefined somatomotor and cerebellar network (SMN and CN) coordinates as seeds, to assess group differences and symptom correlations. Subsequently, we correlated the group-contrast SBC map with existing neurotransmitter maps to explore the neurochemical basis.

**Results:**

In pMDD patients compared to HC, we observed decreased connectivity, especially between the SMN and frontal cortex, within the bilateral SMN, and between the CN and right precentral cortex. Meanwhile, connectivity increased between the SMN and the middle cingulate cortex and between the CN and left precentral cortex in pMDD relative to npMDD and HC. Connectivity between the SMN and angular gyrus was positively correlated with the severity of PmD. Additionally, the aberrant SBC patterns in pMDD were linked to the distribution of dopamine D1 and D2 receptors.

**Conclusions:**

This study provides insights into the aberrant connectivity within the motor circuits and its interactions with non-motor regions in PmD. It also suggests a potential role for dopaminergic dysregulation in the connectivity abnormalities associated with PmD.

## Introduction

Major depressive disorder (MDD) stands as a prominent public health concern affecting an estimated 300 million individuals globally (Evaluation, 2021). Abnormal psychomotor behavior, including slowing movement and thought (retardation) or excessive movement (agitation), is a core feature of MDD (Wuthrich, Nabb, Mittal, Shankman, & Walther, 2022). Studies have revealed that MDD patients with psychomotor disturbance exhibit severe MDD symptom and had a bad response to pharmacological treatments (Bennabi, Vandel, Papaxanthis, Pozzo, & Haffen, 2013; Iwanami et al., 2015). Despite the clinical significance of psychomotor syndrome in MDD, its neural underpinning remains unclear.

Alterations within motor circuits are posited to be associated with psychomotor disturbances (PmD) (Walther & Heckers, 2023). Specifically, the somatomotor network (SMN) and cerebellum, which are integral to the circuit, have been found to associated with psychomotor speed in neuroimaging studies (Guan et al., 2022; Xia et al., 2023). Magioncalda et al. (2020) identified that aberrant connectivity of SMN correlates with psychomotor inhibition in individuals with schizophrenia and bipolar disorder. Meanwhile, a clinical trial using ketamine in patients with MDD revealed that ketamine modulated the cerebellar connectivity during the behavioral inhibition task (Loureiro et al., 2021). Although the motor circuits impairment was observed in patients with PmD (Li, Le, & Jankovic, 2023), the presence of connectivity patterns related to non-motor areas, such as the frontal cortex (Su et al., 2023) and regions within the default mode network (DMN; Conio et al., 2020), suggests that the neural substrates of PmD may not be fully accounted for by the motor circuits hypothesis alone.

An emerging hypothesis posits that non-motor regions, including those within the DMN, also play a role in psychomotor disturbances (Mittal, Bernard, & Northoff, 2017). The term ‘psychomotor’ contains two parts of ‘psycho’ and ‘motor’, suggesting that psychomotor disturbances are abnormal bodily movements precipitated by aberrant mental activity (https://dictionary.apa.org/psychomotor). In a task-based functional magnetic resonance imaging (fMRI) study, Moussa-Tooks et al. (2024) examined brain activity during a psychomotor speed task in individuals with early-onset psychosis, revealing extensive activation in the cerebellum, primary motor areas, and the frontal lobe. Therefore, the interaction between motor and non-motor cortex may reflect this mutual influence between cognition function and motor function (Northoff, Hirjak, Wolf, Magioncalda, & Martino, 2021). A meta-analysis from Hirjak, Kubera, Wolf, and Northoff (2019) synthesized findings from 17 neuroimaging studies on psychomotor disturbances in catatonia, identifying that the atypical frontoparietal network, in conjunction with motor circuits, is implicated in symptom manifestation and is modulated by GABAergic agents. However, research examining the neural substrates of psychomotor disturbances in depression remains relatively scarce.

In summary, we propose that patients with PmD exhibit distinct brain functional connectivity patterns compared to both patients without PmD and healthy controls. Specifically, the differences involve connections within the motor circuit and between the motor circuit and non-motor areas. Furthermore, functional connectivity in patients with PmD is often either the highest or the lowest among the three groups, indicating that patients with PmD are the most severely affected. Moreover, the neurotransmitter disruption underlying the PmD-related connectome may be related to the dopamine system. To test this hypothesis, we examined the functional connectivity anomalies associated with SMN and the cerebellum in psychomotor MDD (pMDD) and non-psychomotor MDD (npMDD), and correlated with their PmD severity. In addition, we correlated the identified aberrant connectivity patterns with a neurotransmitter distribution map to explore the potential neurotransmitter mechanisms at play. The statistical model involved a comparative analysis among MDD patients exhibiting characteristic psychomotor symptoms, those without such symptoms, and a cohort of healthy controls.

## Materials and methods

### Participants

One hundred eighty-four inpatients diagnosed with major depressive disorder according to DSM-5 were recruited for the main dataset from the Department of Depression at Shenzhen Kangning Hospital. The 17-item Hamilton Depression Rating Scale (HAMD) and the 17-item Hamilton Anxiety Rating Scale (HAMA) were administered during the first week of the patients’ hospitalization. The inclusion criteria were as follows: (1) age of 18 years or older, (2) absence of comorbidity with other mental disorders, (3) no neurological conditions, including stroke and substantial head trauma, and (4) no contraindications to MRI scanning. One hundred three healthy controls (HCs) were recruited from the local community. The fMRI data of 34 patients and 9 HCs with excessive head motion, as determined by frame-wise displacement (FD) with mean FD values exceeding 0.25, were excluded. The present study was conducted under the approval by the local institutional review board.

Data collection was completed during the first week of hospitalization to minimize confounding effects of antidepressant use on brain function and avoid potential drug-induced psychomotor disturbances. All patients were subsequently categorized into psychomotor and non-psychomotor subtypes (pMDD and npMDD) based on their psychomotor syndrome. The presence of psychomotor symptoms was determined by assessing item-8 (retardation) and item-9 (agitation) of HAMD. Patients were classified as pMDD if they scored higher than ‘1’ on either item-8 or item-9 (indicating a ‘signature symptom’), with those not meeting this criterion classified as npMDD (Table 1).

**Table 1.** Demographic data for participants

	HC	pMDD	npMDD
	(N = 94)	(N = 107)	(N = 43)
**Gender**			
Female	33 (35.1%)	77 (72.0%)	32 (74.4%)
Male	61 (64.9%)	30 (28.0%)	11 (25.6%)
**Age**			
Mean (SD)	34.4 (11.3)	33.2 (12.7)	34.1 (12.9)
**HAMD**			
Mean (SD)	\	27.9 (6.27)	19.7 (5.35)
Median [min, max]	\	29 [14, 41]	20 [8, 33]
**HAMA**			
Mean (SD)	\	22.4 (7.00)	15.7 (5.25)
Median [min, max]	\	23 [6, 38]	16 [5, 24]
**Illness duration**			
Median	\	6.5 years	6 years

*Note:* In the parenthesis, the percentage represents the proportion out of the total for a specific column. HAMA, Hamilton anxiety rating scale; HAMD, Hamilton depression rating scale; MDD, major depressive disorder; npMDD, MDD patient without psychomotor syndrome; pMDD, MDD patient with psychomotor disturbance; SD, standard deviation.

### MRI data acquisition

MRI data were obtained using a 3.0 Tesla scanner (Discovery MR750 System; General Electric, Milwaukee, WI, USA) with an eight-channel head coil at Shenzhen Kangning Hospital. All participants were instructed to remain still, stay awake with their eyes closed, and not to think about anything. The fMRI data were obtained using an echo-planar imaging (EPI) sequence with the following parameters: repetition time (TR) = 2000 ms, echo time (TE) = 30 ms, flip angle = 90 degree, thickness/gap = 3.5/0.7 mm, acquisition matrix = 64 × 64, field of view (FOV) = 224 mm^2^, 33 axial slices, and 240 time points (8 min). In addition, high-resolution structural MRI scans were obtained using a fast-field echo three-dimensional T1-weighted sequence. The details parameters were as follows: TR/TE = 6.65/2.93 ms, flip angle = 12 degree, acquisition matrix = 256 × 256, FOV = 256 × 256 mm^2^, and 192 sagittal slices with no inter-slice gap.

### Functional data preprocessing

Results included in this article come from analyses performed using CONN (Whitfield-Gabrieli & Nieto-Castanon, 2012) release 22.a and SPM-12 (https://www.fil.ion.ucl.ac.uk/spm/). Functional and anatomical data were preprocessed using a flexible preprocessing pipeline including (1) removal of initial 10 scans, (2) realignment using a six-parameter transformation, (3) slice timing correction, (4) resampled to 3-mm isotropic voxels, (5) MNI-space normalization, and (6) smoothing with a Gaussian kernel of - mm full width at half maximum.

In addition, functional data were denoised, including (1) the regression of potential confounding effects characterized by white matter timeseries, CSF timeseries, 12 motion parameter, and (3) linear trends within each functional run, followed by bandpass frequency filtering of the BOLD timeseries between 0.01 and 0.08 Hz. A detailed preprocessing is presented in the Supplementary Material.

### Seed-based connectivity maps

Seed-based connectivity (SBC) maps were estimated characterizing the spatial pattern of functional connectivity with a seed area. Four seed regions were selected to performed SBC, including left and right lateral somatomotor network (MNI coordinate: left SMN, −55, −12, 29; right SMN, 56, −10, 29), anterior and posterior cerebellar network (MNI coordinate: aCereb, 0, −63, −30; pCereb, 0, −79, −32) regions of interest (ROIs). These networks were defined in 32 HPC-ICA network atlas (Nieto-Castanon, 2020). We selected the lateral SMN seeds over the medial seeds due to their accessibility for transcranial magnetic stimulation, which has significant clinical relevance.

Functional connectivity strength was represented by Fisher-transformed bivariate correlation coefficients from a weighted general linear model, estimated separately for each seed area and target voxel, modeling the association between their BOLD signal timeseries. To compensate for possible transient magnetization effects at the beginning of each run, individual scans were weighted by a step function convolved with an SPM canonical hemodynamic response function and rectified.

### Neurotransmitter system association

To determine whether the abnormal connection patterns were correlated with specific neurotransmitter systems, we performed an analysis of the spatial correlation between the connectivity map and the distribution patterns of various receptor/transporter systems using the JuSpace software (https://github.com/juryxy/JuSpace) (Dukart et al., 2020). The distribution of neurotransmitter receptors was obtained from previous study, measured by positron emission tomography (PET). We focused on two serotonin receptors, the 5-hydroxytryptamine 1a (5-HT1a) and 5-HT2a, two dopamine receptors, the D1 and D2, and the gamma-aminobutyric acid (GABA) receptor. The analysis involved the following steps: (1) estimating the effect size (Cohen’s d) maps from the t-tests comparing all seed-based functional connectivity between pMDD and HC, as well as between pMDD and npMDD; (2) downsampling the voxel-wise neurotransmitter and effect size maps into 119 regions by averaging the voxel values within each region using a neuromorphometrics atlas which derived from the ‘MICCAI 2012 Grand Challenge and Workshop on Multi-Atlas Labeling’ (https://masi.vuse.vanderbilt.edu/workshop2012/index.php/Challenge_Details); (3) applying Pearson’s correlation to assess the association between the effect size map and the neurotransmitter map; and (4) correcting the *p* values using the false discovery rate (FDR) method, adjusted for the number of neurotransmitter receptors. A *p* value of less than 0.05 after FDR correction was considered statistically significant.

### Statistical analysis

#### Clinical profile between subtypes of MDD

We examined the differences in age and gender between the pMDD and npMDD groups using t-tests and chi-square tests. To characterize the clinical features of each subtype, we compared the depression and anxiety symptoms between the two subgroups based on the total scores of HAMD and HAMA using t-tests. Additionally, we evaluated item-level differences in HAMD scores between the subtypes using a one-way ANOVA, followed by post-hoc analysis. Age and gender were incorporated as covariates in the model.

#### Group-level analyses of the brain metrices

Group-level analyses of SBC were performed using the General Linear Model (GLM). Two GLMs of interest were identified, including (1) an ANOVA model for examining the SBC differences among HC, pMDD, and npMDD and (2) a regression model for predicting the SBC based on the MDD patients’ psychomotor score on the HAMD - 17. These two models incorporated age and gender as covariates to mitigate the effect of demographic factors on the outcomes. The psychomotor score was calculated as the sum of the scores for items 8 and 9.

Considering that patients exhibiting characteristic psychomotor disturbances also presented with pronounced depressive symptoms, we conducted a controlled analysis to test whether the observed effects were potentially confounded by the severity of depression. To this end, patients were re-grouped based on their HAMD scores, with those scoring above 24 classified as having severe MDD (sMDD), in accordance with established cutoff criteria (Carrozzino, Patierno, Fava, & Guidi, 2020), and the remainder categorized as having moderate MDD (mMDD). Then, two control GLMs were specified to evaluate the difference in SBC among HC and two MDD subgroups.

Voxel-level hypotheses were evaluated using multivariate parametric statistics with random-effects across subjects and sample covariance estimation across multiple measurements. Inferences were performed at the level of individual clusters (groups of contiguous voxels). Cluster-level inferences were based on parametric statistics from Gaussian Random Field theory (Worsley et al., 1996). Results were thresholded using a combination of a cluster-forming *p* < 0.001 voxel-level threshold, and a family-wise corrected p-FDR < 0.05 cluster-size threshold.

#### Post-hoc analyses of the ANOVA models

A post-hoc analysis was conducted when any ANOVA model showed significant group difference. Specifically, the pair-wise comparison was performed to identify the exact difference between groups by using t-test and the *p* value was corrected by the FDR method.

## Results

### Clinical profile of MDD patients with psychomotor disturbance

**Figure 1.**
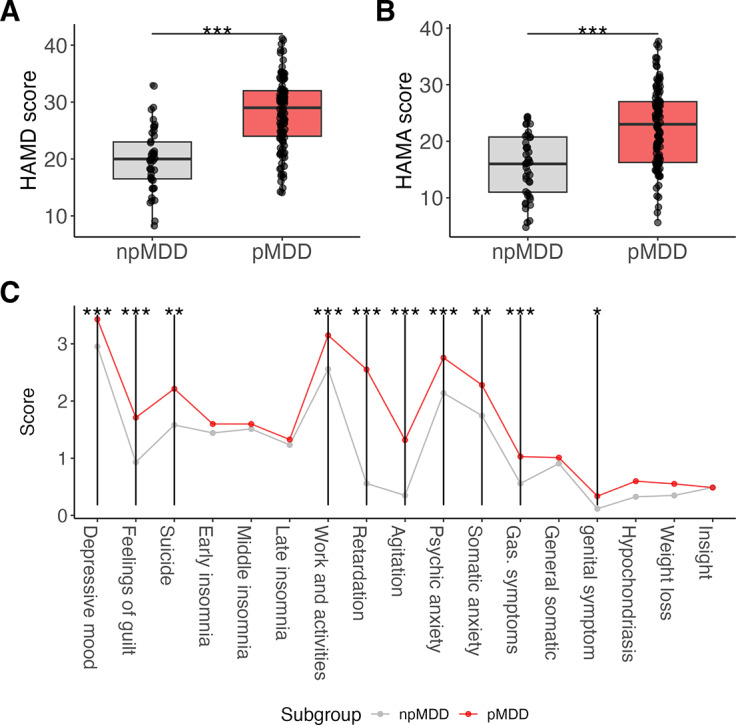
Clinical profile for the subgroups of the patients. The panels depict group differences in (A) the severity of depressive symptoms, (B) anxiety levels, and (C) the presence of 17 specific symptoms among patients with and without psychomotor disturbance. Depression severity was quantified using the 17-item Hamilton Depression Rating Scale, while anxiety levels were assessed with the Hamilton Anxiety Rating Scale. *p < 0.05, **p < 0.01, ***p < 0.001. Note: HAMA, Hamilton Anxiety Rating Scale; HAMD, the Hamilton depression rating scale; pMDD and npMDD: major depressive disorder with and without significant psychomotor disturbance.

Among the 107 patients identified with the psychomotor subtype, 58 exhibited significant retardation, 7 showed significant agitation, and 42 had both conditions. There were no significant differences in age or gender between the pMDD and npMDD (age: *t* = −0.383, *p* = 0.702; gender: χ^2^ = 0.011, *p* = 0.918). The HAMD total scores were significantly higher in the pMDD compared to the npMDD (*t* = 7.683, *p* < 0.001, Figure 1A), as were the HAMA total score (*t* = 6.035, *p* < 0.001, Figure 1B). Additionally, a significant interaction effect between subtype and item-level HAMD scores was identified in the ANOVA model (*F*(11,1476) = 11.869, *p* < 0.001). Figure 1C presents the post-hoc analysis results, which revealed significantly higher scores in specific item-level assessments (all *pFDR* < 0.05), including depressive mood (item 1), guilt feeling (item 2), suicidality (item 3), retardation (item 8), agitation (item 9), psychic anxiety (item 10), somatic anxiety (item 11), appetite decrease(item 12), sexual interest (item 13), and hypochondriasis (item 14).

### Somatomotor network dysfunction in psychomotor subgroup

**Figure 2.**
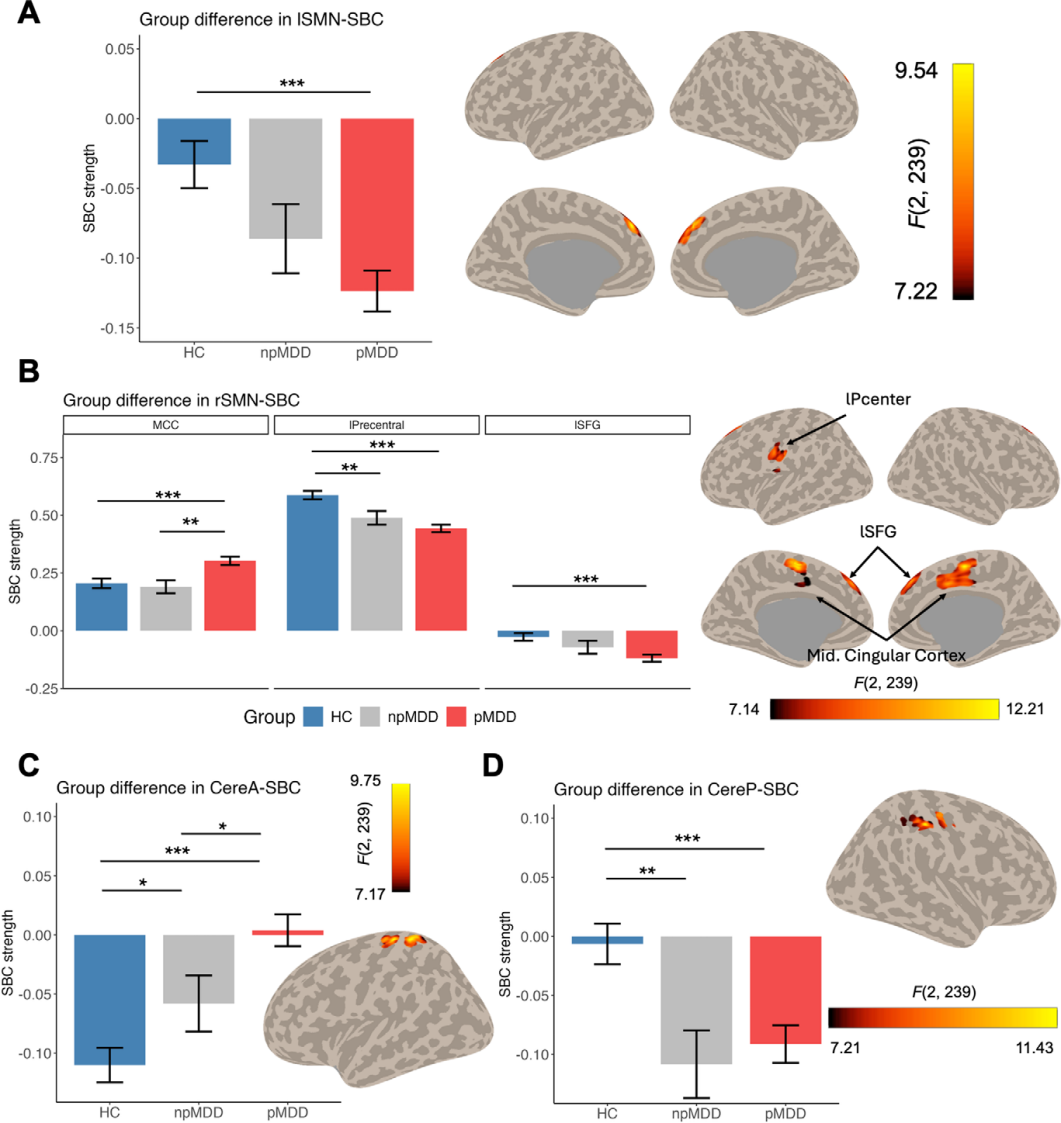
Functional connectivity alteration in psychomotor subtype. The panels show the disparities among patient subgroups and healthy controls in terms of seed-based connectivity (SBC) strength, with a focus on the seeds of the (A) left somatomotor network (lSMN), (B) right somatomotor network (rSMN), (C) anterior cerebellar network (CereA), and (D) posterior cerebellar network (CereP). The brain plots highlight the regions of significant clusters identified through analysis of variance (ANOVA), and the bar plots depict the outcomes of post-hoc multiple comparisons. *p < 0.05, **p < 0.01, ***p < 0.001. Note: FDR, false-discovery rate correction; lPcentral, left precentral gyrus; lSFG, left superior frontal gyrus; MCC, middle cingulate cortex.

For the left SMN SBC, GLM identified a significant group effect of a cluster on the right superior frontal gyrus (SFG, *F*(2,239) = 11.69, *pFDR* < 0.001, Fig. 2B). Post-hoc analysis indicated a decreased FC in pMDD compared to HC (*t* = −4.059, *pFDR* < 0.001). Although FC in pMDD was also lower than in npMDD, this decrease was not statistically significant (*t* = −1.312, *pFDR* = 0.191). Non-significant result was found between npMDD and HC (*t* = −1.830, *pFDR* = 0.103).

The GLM identified three clusters exhibited significant group effects for the right SMN SBC (Figure 2C), including the middle cingulate cortex (MCC, *F*(2,239) = 17.22, *pFDR* < 0.001), the left precentral gyrus (*F*(2,239) = 12.01, *pFDR* < 0.001) and the left SFG (*F*(2,239) = 14.48, *pFDR* < 0.001). Notably, increased connectivity in the MCC was observed in the pMDD group compared to both the HC and npMDD groups (pMDD versus HC: *t* = 3.559, *pFDR* = 0.001; pMDD versus npMDD: *t* = 3.214, *pFDR* = 0.002). Additionally, decreased connectivity was observed in the left precentral cortex (*t* = −5.817, *pFDR* < 0.001) and the left SFG in pMDD when compared to HC (*t* = −3.972, *pFDR* < 0.001). Nevertheless, a significant reduction in connectivity within the left precentral gyrus was also observed between the npMDD and HC (*t* = −5.817, *pFDR* < 0.001).

### Cerebellum connectivity in psychomotor subgroup

Figure 2D illustrates the group differences in functional connectivity associated with the anterior cerebellum seed. A cluster in the left postcentral gyrus was identified in response to group effects (*F*(2,239) = 13.11, *pFDR* < 0.001). Post-hoc analysis revealed a significant increase FC in pMDD compared to the other two groups (pMDD versus HC: *t* = 5.631, *pFDR* < 0.001; pMDD versus npMDD: *t* = 2.394, *pFDR* = 0.026). Additionally, a significant increase of the connectivity was detected in npMDD than HC (*t* = 1.975, *pFDR* = 0.049).

Regarding the posterior seed of the cerebellar network, the GLM detected one significant cluster in the right postcentral gyrus (*F*(2,239) = 11.58, *pFDR* < 0.001). Specifically, connectivity was diminished in both pMDD and npMDD when contrasted with HC (pMDD versus HC: *t* = −3.535, *pFDR* = 0.001; npMDD versus HC: *t* = −3.261, *pFDR* = 0.002).

To assess the robustness of the SBC group-comparison findings, we conducted two supplementary analyses (see supplementary material). In the first analysis, we repeated the primary comparison process while excluding data from patients with significant agitation. In the second analysis, we incorporated head motion as a covariate in the models. Both analyses consistently replicated the main findings, confirming the robustness of our results (see Supplementary Figures S1 and S2).

### Connectivity Symptom correlation

**Figure 3.**
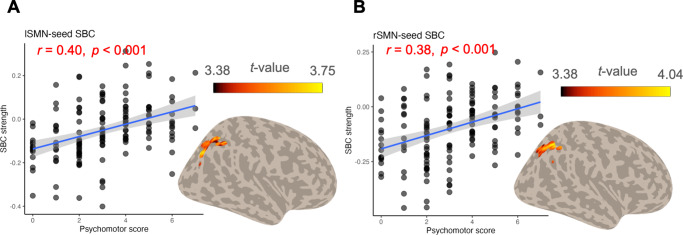
Brain-symptom correlation map. The panels depict Pearson’s correlation coefficients between the severity of psychomotor symptoms and seed-based connectivity (SBC), with a focus on the seeds of both the left (lSMN, A) and right (rSMN, B) somatomotor networks. The brain plot identifies the locations of significant clusters derived from the correlation analysis, while the dot plot illustrates the pattern of coupling between the two variables. Psychomotor severity was quantified by summing the scores of psychomotor-related items from the Hamilton depression rating scale.

Figure 3 shows the correlation from the seed-based connectivity and psychomotor symptom. For the left SMN SBC, a significant positive correlation was observed between connectivity to the right angular gyrus and the severity of psychomotor symptoms (*r* = 0.401, *p* < 0.001, Figure 3A). Additionally, connectivity between the right SMN and the right angular gyrus was also found to correlate with the psychomotor syndrome (*r* = 0.381, *p* < 0.001, Figure 3B). However, no significant correlations were detected for the cerebellum-seed connectivity with psychomotor symptoms.

### Correlations between connectivity and PET maps

**Figure 4.**
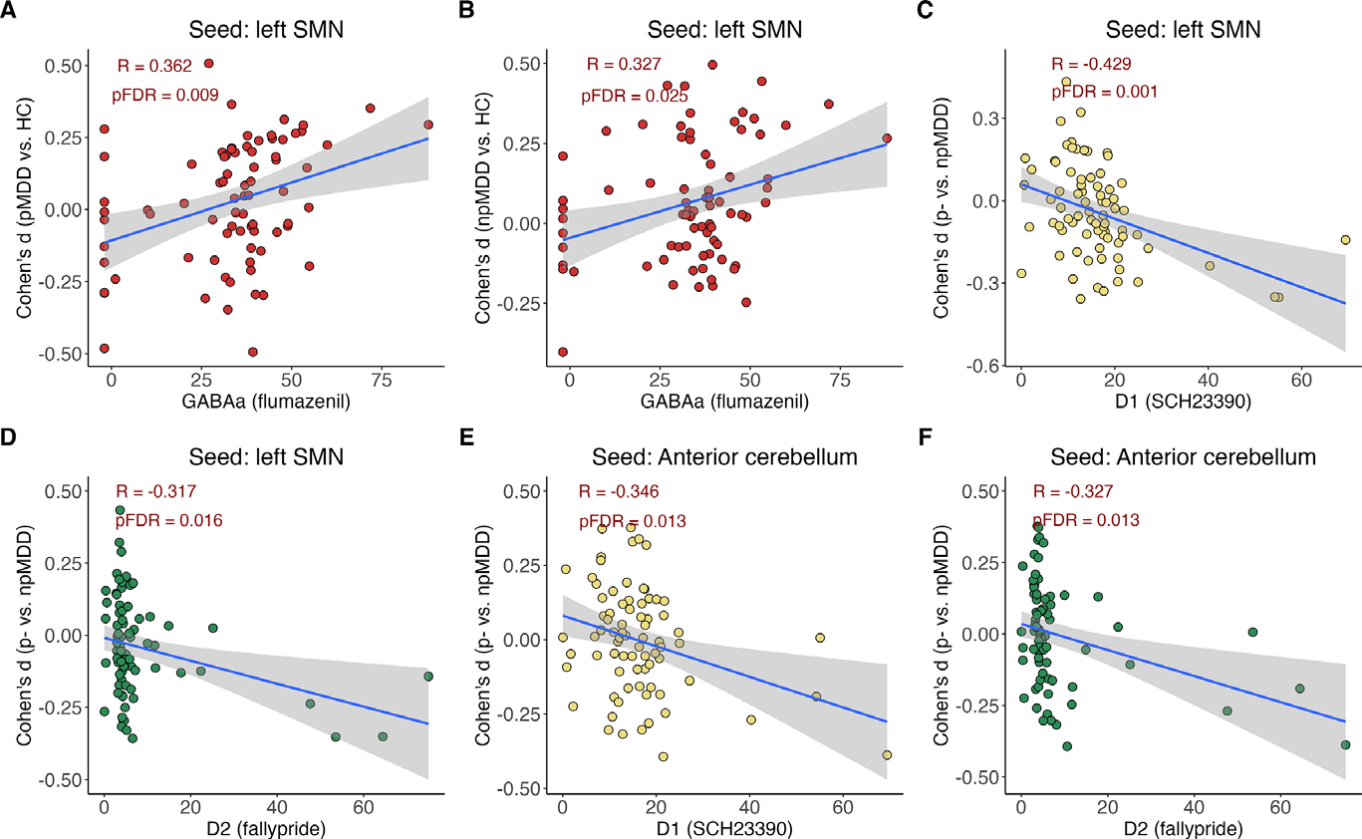
The overlap between functional alteration patterns and neurotransmitter distributions. (A and B) The correlation between the distribution of GABAa receptors and the seed-based connectivity (SBC) of the left somatomotor network (lSMN) for the psychomotor subgroups compared to healthy controls. (C and D) The correlation between the distributions of D1 and D2 receptors and the lSMN SBC map when contrasting the two patient subgroups. (E and F) The correlation between the distributions of D1 and D2 receptors and the anterior cerebellum SBC map for the same patient subgroup comparison. The PET tracers used for the neurotransmitter distributions are indicated in parentheses.

Figure 4 demonstrates the correlation between connectivity pattern and distribution of neurotransmitter receptors. For the left SMN seed, we found a positive correlation between the SBC differences in pMDD compared to HC and the distribution of GABA (*r* = 0.362, *pFDR* = 0.009, Figure 4A). This correlation was also evident in the comparison between npMDD and HC (*r* = 0.327, *pFDR* = 0.025, Figure 4B). Furthermore, using the same seed, a significant negative correlation was observed between the SBC differences across two subgroups and the dopamine D1 and D2 receptors (D1: *r* = −0.492, *pFDR* = 0.001; D2: *r* = −0.317, *pFDR* = 0.016, Figure 4C and D). A similar negative correlation was identified in the anterior cerebellum seed-based connectivity, where the subgroup difference in connectivity was associated with the dopamine D1 and D2 receptors (D1: *r* = −0.346, *pFDR* = 0.013; D2: *r* = −0.327, *pFDR* = 0.013, Figure 4E and F). However, no significant correlations were detected for the right SMA or the posterior cerebellum seed-based connectivity.

### Control analysis with depression severity subgroups

**Figure 5.**
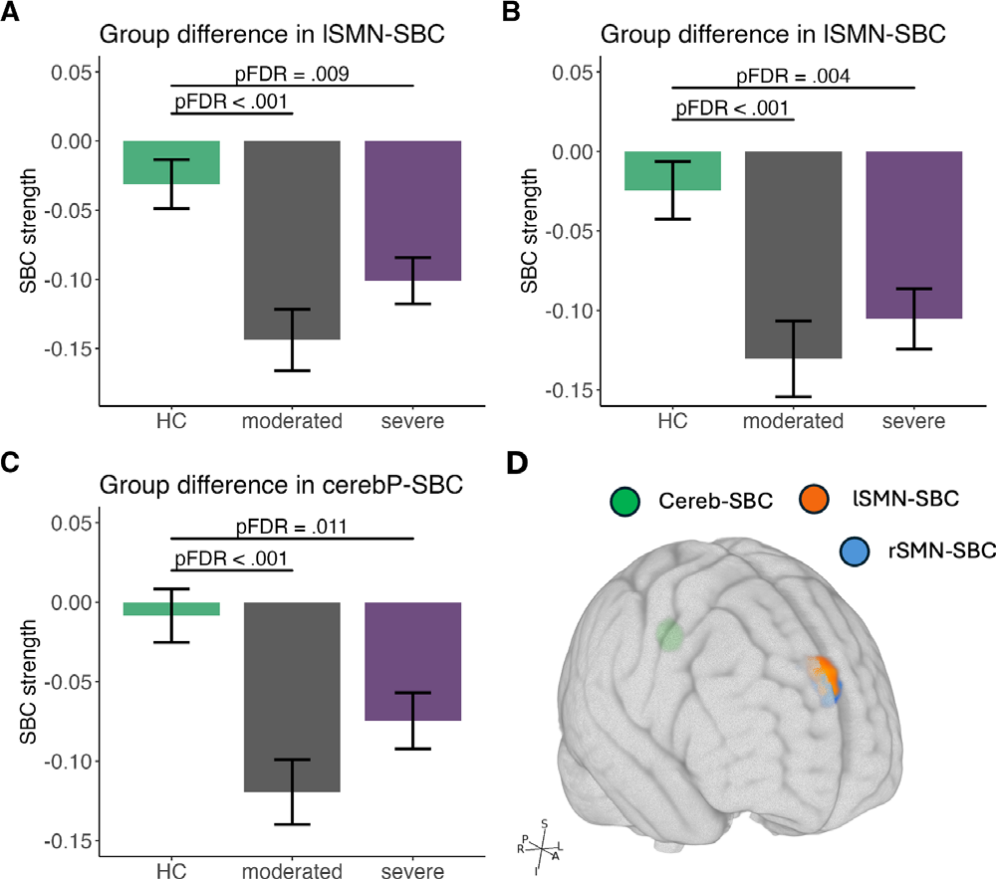
Functional dysregulation in the control analysis. The panels show significant clusters resulting from group comparisons of seed-based functional connectivity (SBC) strength among patients with moderate to severe depression symptoms and healthy controls. Significant clusters were identified in the SBC associated with the (A) left somatomotor network (lSMN) seed, (B) right somatomotor network (rSMN) seed, and (C) posterior cerebellar network (CerebP) seed. The brain plot in (D) provides a visual representation of the locations of these significant clusters, while the bar plots in (A)–(C) depict the results of post-hoc comparisons among the three groups.

Figure 5 illustrates the results for control analysis. Specifically, in the left SMN-seed SBC, a significant cluster was identified within SFG (*F*(2,239) = 11.87, *pFDR* < 0.001). Post-hoc analysis revealed reduced connectivity in moderated MDD compared to HC (*t* = −4.175, *pFDR* < 0.001, Figure 5A), as well as in severe MDD compared to HC (*t* = −2.777, *pFDR* = 0.009, Figure 5A). A similar significant cluster in the SFG for the right SMN-seed SBC (*F*(2,239) = 11.38, *pFDR* < 0.001), with decreased connectivity observed in both moderate and severe MDD relative to HC (moderated MDD versus HC: *t* = −3.651, *pFDR* < 0.001; severe MDD versus HC: *t* = −3.001, *pFDR* = 0.004, Figure 5B). Additionally, a significant cluster was detected in the right precentral cortex with the posterior cerebellum-seeded SBC (*F*(2,239) = 12.27, *pFDR* < 0.001), where a significant reduction in connectivity was noted between severe MDD and HC (*t* = −4.206, *pFDR* < 0.001, Figure 5C), and moderate MDD and HC (*t* = −2.703, *pFDR* = 0.01, Figure 5C). However, no significant clusters were identified with the anterior cerebellum seed-SBC. Moreover, no significant correlations were found between the SBC measures and the HAMD total scores.

## Discussion

In this study, we concentrated on delineating the aberrant functional connectivity patterns of the SMN and cerebellum in MDD patients with psychomotor disturbances. Clinically, pMDD demonstrated elevated levels of depression and anxiety compared to those without significant disturbances (npMDD), yet no significant differences were observed in demographic profiles. Neuroimaging findings revealed alterations in both motor network and the interaction between motor and non-motor network in pMDD. Notably, reduced connectivity was identified between the motor cortex and SFG, as well as bilateral connections of precentral cortex, and the posterior cerebellum to the motor cortex in pMDD relative to HC. Conversely, an oincrease in connectivity was noted between the motor cortex and the cingulate gyrus, and between the anterior cerebellum and the motor cortex in pMDD compared to HC. Additionally, an association was established between motor cortex to the angular gyrus SBC and the severity of psychomotor symptoms. Finally, the aberrant connectivity patterns observed in the motor cortex and cerebellum were correlated with the distribution of GABA, and dopamine receptors D1 and D2 in the brain.

### Clinical profile of psychomotor subtype

In our dataset, 71.3% of the MDD patients were identified as psychomotor subtype. The proportion aligns with the previous study which reported that approximately 70% of MDD patients exhibit psychomotor syndrome (Wuthrich et al., 2023), confirming its prevalence in clinical settings as noted by van Loo, de Jonge, Romeijn, Kessler, and Schoevers (2012). The depressive symptom in pMDD exhibited higher than npMDD. Symptom-level comparisons revealed that pMDD patients experienced more severe symptoms, including suicidality, somatic complaints, and anxiety. These findings underscore the notion that pMDD represents a particularly severe subtype within the broader MDD population (Wuthrich et al., 2023).

### Connectivity aberrance in the motor circuits

We identified the connection alteration within the bilateral SMN and between the cerebellum and SMN in pMDD. Similar alterations in the SBC of the bilateral primary motor cortex have been found in previous research (Wuthrich et al., 2023). This change may stem from disruptions in the white matter motor pathways that connect the left and right motor cortices (Al-Sharif, Zavaliangos-Petropulu, & Narr, 2024). Bingham et al (2023) used diffusion-weighted MRI to demonstrate that tractography of the corpus callosum correlates with psychomotor disturbances in MDD. Furthermore, study suggests that alterations in white matter fibers are associated with hypodopaminergic states (Shankman, Mittal, & Walther, 2020).

The cortico-cerebellar loop, composed of the connections between cerebellar and motor areas, is known to engaged in the fine-tuning of motor performance (Dahms, Brodoehl, Witte, & Klingner, 2020). The impairment of this loop was reported to multiple psychiatric conditions with motor differences, like Parkinson’s disease (Pietracupa et al., 2024), schizophrenia (Lefebvre et al., 2024), and MDD (Wuthrich et al., 2023). The increased cerebellum-seed connectivity may suggest a higher inhibition between the cerebellum and motor cortex (Lefebvre et al., 2024).

### Functional dysregulation for the interaction between the motor circuits and non-motor areas

For SBC with bilateral SMN seeds, we found a decreased connectivity to the frontal region and an increased connectivity to the MCC. These results demonstrated that the abnormal connections had not only come out in the intra-connectivity within SMN, also the inter-connectivity beyond motor area. The SFG, which was reported to be involved in the microcircuit of visuomotor (McCarthy, 2021) and motor functions (Gao et al., 2018; Martino et al., 2011), was found to be dysfunction in psychosis with psychomotor disturbance (Moussa-Tooks et al., 2024). Meanwhile, a lesion study shown that SFG lesion was related to the impairment of patient’s working memory (du Boisgueheneuc et al., 2006), suggesting that the amorality between SMN-SFG connectivity may explain the cognitive impairment in patients with PmD (Bennabi et al., 2013). In summary, the SFG functions in either cognitive or motor domain (Li et al., 2013), corresponding to the psychomotor disturbance which including a ‘psych’ (cognition) and a ‘motor’ aspects (Walther & Heckers, 2023).

The connectivity between MCC and motor area was reported in the previous study (van Hout, van Heukelum, Rushworth, Grandjean, & Mars, 2024), and the abnormal activity of MCC is critical to the movement disorders, like Tourette syndrome and attention deficit hyperactivity disorder (Vogt, 2016). Previous review (Caruana et al., 2018) summarized that the function of MCC convergence into two lines: one from the motor domain, showing that MCC was involved in movement monitoring (Procyk et al., 2016); the other from the affective domain, arguing that MCC was engaging into the negative affect response (Shackman et al., 2011). Therefore, it is believed to play a critical role in diagnoses for MDD (Yan, 2024). Furthermore, the opposite connection direction between SMN-SFG and SMN-MCC may be due to the functional antagonism between DMN and executive control network (Menon, 2023).

The SBC between SMN and angular gyrus was found to correlate with the severity of patients’ psychomotor syndromes. A previous study has established that the angular gyrus’s connectivity with the motor cortex plays a crucial role in the execution of fine hand movements (Baarbé et al., 2021). Additionally, the angular gyrus is a node within the DMN, and the interaction between the DMN and SMN is intricately linked to motor symptom (Northoff et al., 2021). For instance, disruptions in the SMN-DMN connectivity have been observed in older adults experiencing motor disturbances (Rodriguez-Sabate, Morales, Sanchez, & Rodriguez, 2019). From a review paper, Conio et al. (2020) have suggested that an imbalance in neuronal activity between the DMN and SMN, potentially resulting from abnormal neurotransmitter transfer, could serve as a neural signature for psychomotor symptoms. Our findings, which indicate a positive correlation between the strength of this connectivity (SBC) and symptom severity, might imply an over-dominance of the DMN over the SMN, culminating in abnormal movement patterns.

### Neurotransmitter receptor underlying connectivity alteration

The conjunction analysis between SBC map and neurotransmitter distributions revealed an overlap between the connectivity abnormalities and the GABA and dopamine receptor distribution, while the correlation to dopamine receptor was specifically to the psychomotor subtype. The previous study had shown that the participant will have poorer motor-skill learning rate when blocking the dopamine receptor function (Curtin, Taylor, Bellgrove, Chong, & Coxon, 2024). Meanwhile, the disruption of the dopaminergic system is considered as the pathology to the movement disturbances in psychosis (Mittal et al., 2017). A large sample study measured the dopaminergic innervation by single-photon emission computed tomography dopamine transporter imaging in patients with Parkinson’s disease and comorbid by depression, and they found that the motivational symptoms of the patient were associated with the dopaminergic neurodegeneration in striatum (Costello, Schrag, Howard, & Roiser, 2024).

### Connectivity displayed a progressive gradient of changes in psychomotor subtype

While group differences were also present in the control analysis, a key finding is the distinct gradient in connectivity changes observed within the psychomotor subgroups. Specifically, the decrease in connectivity seen in the npMDD group was even more pronounced in the pMDD group, with a converse pattern emerging for increased connectivity. This gradient pattern was notably absent in the moderate and severe depression severity subgroups, strengthening the evidence that the observed alterations in subgenual cingulate cortex (SBC) connectivity are likely specific to the psychomotor syndrome. Consequently, patients with psychomotor disturbance (PmD) may require different treatment strategies than those without PmD. The cingulate cortex and cerebellum – identified in this study as uniquely abnormal regions in PmD – represent potential neuromodulation targets, such as transcranial magnetic stimulation.

This study has several limitations. First, psychomotor disturbance was assessed using a single 5-point Likert scale item from the HAMD-17, which offers a rudimentary evaluation lacking nuance. Future research should adopt more comprehensive tools, such as Parker and McCraw’s (2017) specialized questionnaire or behavioral tasks accessing motor speed (e.g. the nine-hole task; (Moussa-Tooks et al., 2024). Second, the psychomotor subtype classification relied solely on the presence of agitation or retardation. Although both are manifestations of psychomotor disturbance, emerging evidence suggests distinct neural underpinnings (Mittal et al., 2017), including differentiated dynamic brain activity patterns (Martino et al., 2016). Future studies should aim to delve deeper into this area to elucidate the distinct neural markers associated with agitation and retardation. Future work should explicitly disentangle the neural correlates of agitation versus retardation. Third, as our cohort comprised remitted MDD patients, the impact of illness duration on brain activity remains unclear. Future investigations should account for illness chronicity or validate our findings in first-episode, drug-naïve cohorts to minimize confounding effects. Finally, neurotransmitter correlations were inferred indirectly via atlas-based PET data comparisons. While this approach provides preliminary mechanistic insights rather than definitive evidence, it establishes a foundational framework for investigating neurotransmitter pathology in psychotic psychomotor disturbances.

In conclusion, this study identifies connectome-based neural underpinnings of psychomotor disturbance, revealing that connectivity between the right somatomotor network and midcingulate cortex, along with cerebellar-motor pathways, may represent potential biomarkers for psychomotor subtyping. Furthermore, these aberrant connectivity patterns implicate disruptions in dopaminergic transmission. Future research should validate our findings across psychiatric contexts with prominent psychomotor features – such as schizophrenia – and elucidate underlying neurotransmitter pathophysiology.

## Supporting information

Liang et al. supplementary material Liang et al. supplementary material

## Abbreviations

DMN: default mode network
EPI: echo-planar imaging
FD: frame-wise displacement
FOV: field of view
fMRI: functional magnetic resonance imaging
HAMA: the Hamilton Anxiety Rating Scale
HAMD: Hamilton depression rating scale
MDD: major depressive disorders
QC: quality control
TE: echo time
TR: repetition time

## Supplementary Material

The supplementary material for this article can be found at http://doi.org/10.1017/S0033291725101852.
