# Supplementary material for: Motor circuits and beyond: Functional connectivity related to psychomotor syndromes in depression
Source: Psychol Med. 2025 Sep 29;55:e285. doi: 10.1017/S0033291725101852 (PMC12527541; doi:10.1017/S0033291725101852)
Supplement: Liang et al. supplementary material [file S0033291725101852sup001.docx]

**Motor circuits and beyond: functional connectivity related to psychomotor syndromes in depression**

**Robustness Analysis 1: Group-level functional connectivity analysis excluding agitated patients**

The psychomotor subtype (pMDD) composed of the patients with retardation (n = 100) or agitation (n = 7). To evaluate whether our primary group-level results were substantially influenced by psychomotor subtypes, we constructed four ANOVA models examining seed-based connectivity (SBC) differences after excluding data from the seven agitated patients. These models corresponded to the four seeds used in the main analysis.

All ANOVA models revealed significant group differences (all *p* < 0.001). Specifically, significant SBC variations were identified for the left and right somatomotor network (SMN) seeds (left SMN: *F* = 11.102, *p* < 0.001; right SMN: *F* = 16.915, *p* < 0.001), as well as the anterior and posterior cerebellar network seeds (anterior seed: *F* = 13.623, *p* < 0.001; Posterior seed: *F* = 22.562, *p* < 0.001). Subsequently, we performed FDR-corrected post-hoc pairwise comparisons for each model. Results are illustrated in Figure S1.

This analysis confirmed that our main findings were not affected by the influence of psychomotor heterogeneity.

**Robustness Analysis 2: Group-level functional connectivity analysis with motion artifact regression**

To determine whether head motion influenced our main findings, we constructed four ANOVA models assessing group differences in seed-based connectivity (SBC). These models employed strict nuisance regression controlling strategy than the model used in the main text, including age, gender, and head motion (using mean framewise displacement).

All models demonstrated significant group effects (all p < 0.001). Specifically, significant SBC differences emerged among the three groups for left and right somatomotor network (left SMN: *F* = 10.841, *p* < 0.001; right SMN: *F* = 15.356, *p* < 0.001), and anterior and posterior cerebellar network (anterior seed: *F* = 12.821, *p* < 0.001; Posterior seed: *F* = 23.975, *p* < 0.001). We subsequently conducted pairwise post-hoc comparisons with false discovery rate (FDR) correction for each model, separately. Results are presented in Figure S2.

This analysis indicates that our main findings remain robust when controlling for multiple nuisance covariates.

**Supplementary Figures**


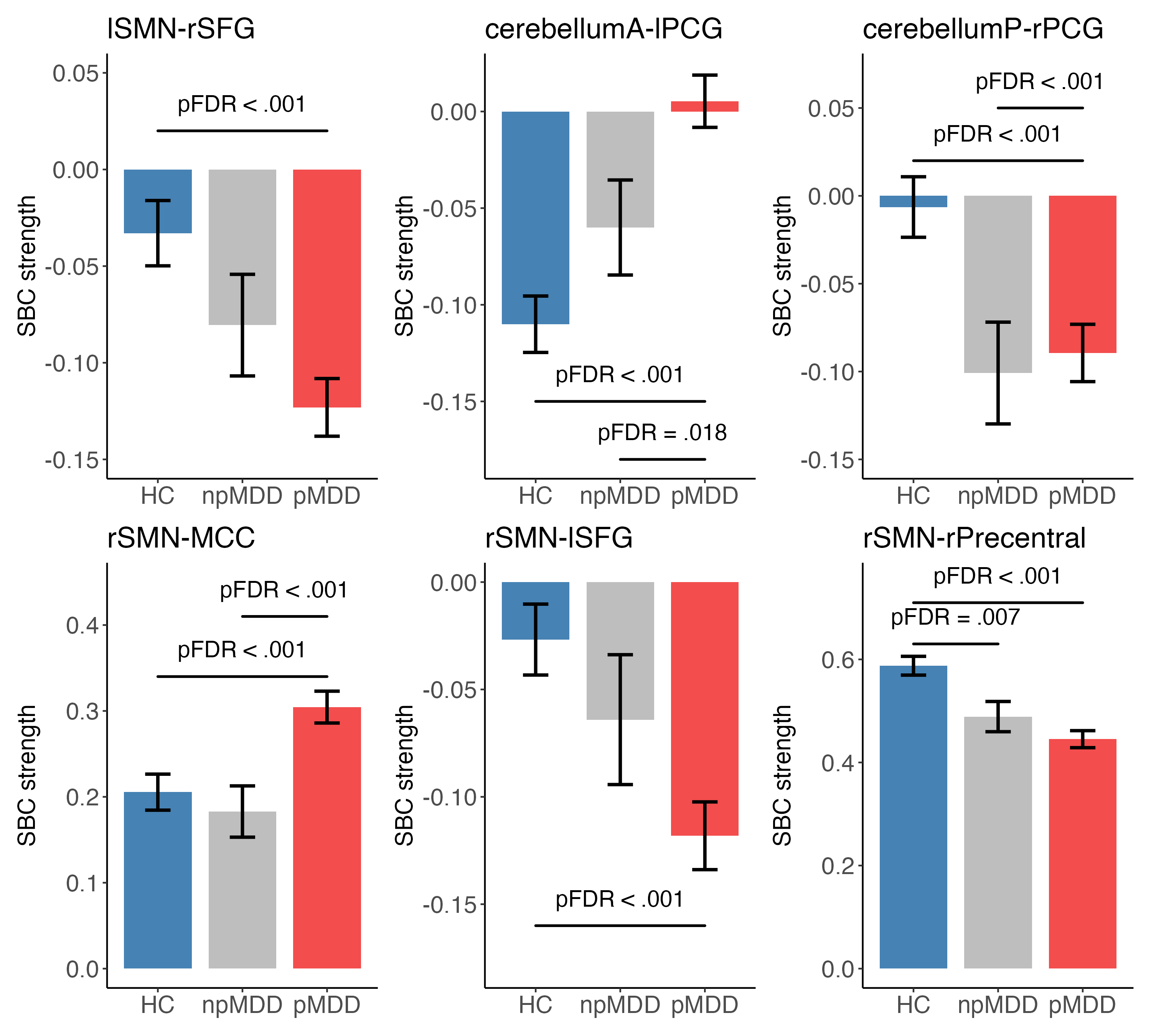


**Figure S1. Seed-based connectivity (SBC) group differences after excluding agitation patients** Pairwise ANOVA results for SBC differences, corresponding to Figure 2 (main text). Bar plots show connectivity strength for: (A) SMN seeds (left/right somatomotor network: lSMN/rSMN), and (B) Cerebellar seeds (anterior/posterior cerebellum: CerebA/CerebP). All group differences remained significant (pFDR < 0.001). Regions: lSFG/rSFG = left/right superior frontal gyrus; lPCG/rPCG = left/right postcentral gyrus; rPrecentral = right precentral gyrus; MCC = midcingulate cortex.


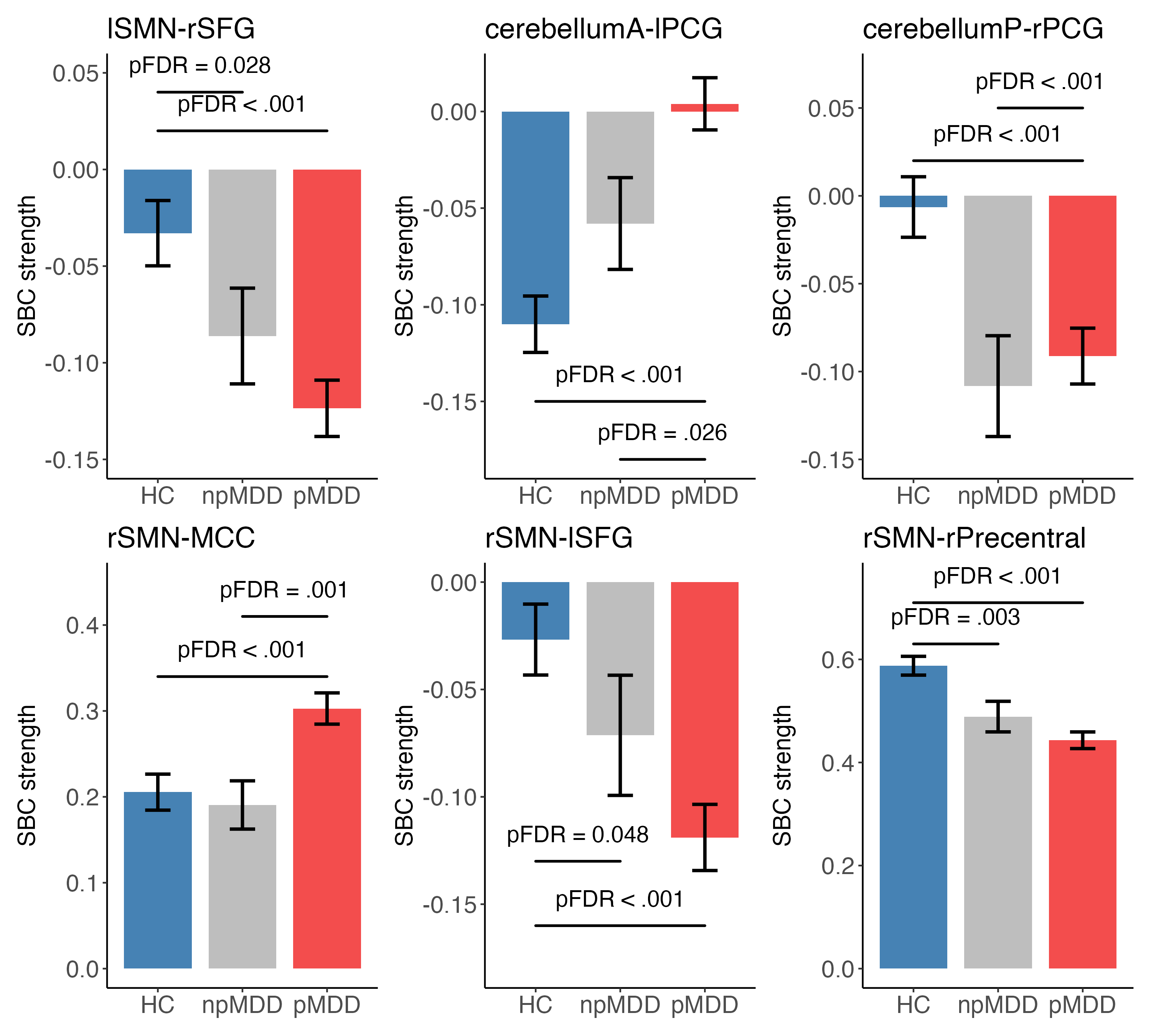


**Figure S2 Seed-based connectivity (SBC) group differences after motion regression** Pairwise ANOVA results for SBC differences, corresponding to Figure 2 (main text). Bar plots show connectivity strength for: (A) SMN seeds (left/right somatomotor network: lSMN/rSMN), and (B) Cerebellar seeds (anterior/posterior cerebellum: CerebA/CerebP). All group differences remained significant (pFDR < 0.001). Regions: lSFG/rSFG = left/right superior frontal gyrus; lPCG/rPCG = left/right postcentral gyrus; rPrecentral = right precentral gyrus; MCC = midcingulate cortex.
